# 5-[(1,3-Dimethyl-5-oxo-2-sulfanylideneimidazolidin-4-yl­idene)amino]-2-methyl­isoindoline-1,3-dione

**DOI:** 10.1107/S2414314621003229

**Published:** 2021-04-09

**Authors:** Sambasivarao Kotha, Naveen Kumar Gupta, Saima Ansari

**Affiliations:** a Department of Chemistry, Indian Institute of Technology Bombay, Powai, Mumbai - 400076, India; University of Aberdeen, Scotland

**Keywords:** crystal structure, deep eutectic mixture, thio­hydantoin, pthalamide, dimethyl thio­urea

## Abstract

The title *N*,*N*-di­methyl­thio­hydantoin containing an *N*-methyl­ated pthalimide group, C_14_H_12_N_4_O_3_S, arose from an unexpected reaction in a deep eutectic di­methyl­thio­urea–tartaric acid solvent system. The mean planes of the ring systems are twisted at an angle of 73.84 (17)°. In the crystal, weak C—H⋯O hydrogen bonds connect the mol­ecules.

## Abstract 2

The title pthalamide-substituted thio­hydantoin arose from an unexpected reaction in a deep eutectic di­methyl­thio­urea–tartaric acid solvent system.

## Graphical abstract

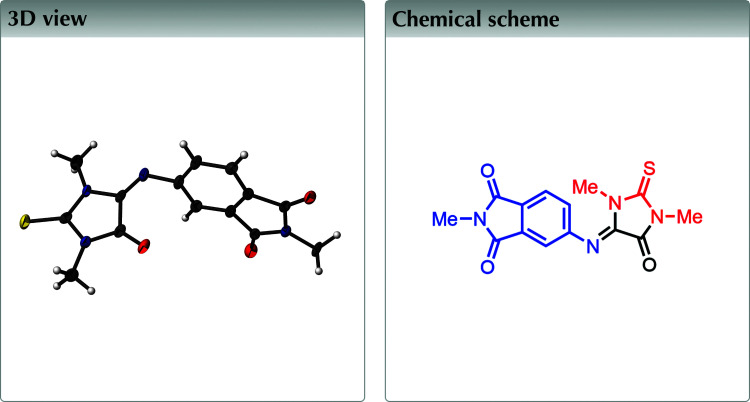

## Structure description

Thio­hydantoins are effective in treating various biological disorders (Spicer *et al.*, 2013 ▸; Wang *et al.*, 2021 ▸; Huang *et al.*, 2018 ▸; Manzanaro *et al.*, 2006 ▸). In an attempt to synthesize 5-amino-substituted hydantoins and thio­hydantoins (Kotha *et al.*, 2019 ▸), we unexpectedly obtained the title imino-substituted thio­hydantoin **1**.

**Figure 1:**
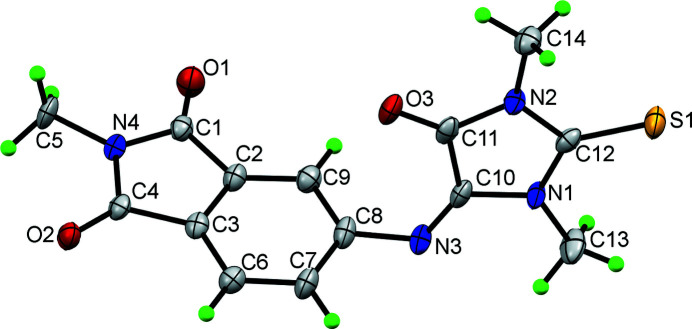
The mol­ecular structure of **1**, showing 50% displacement ellipsoids.

The ^1^H NMR spectrum confirmed the absence of two H atoms (CH—NH grouping) and the ^13^C spectrum showed the downfield shift for the carbon atom of the C—N bond. To establish its structure unambiguously, the crystal structure was determined, which confirmed the presence of the C10=N3 double bond [1.252 (4) Å] (Fig. 1 ▸). The remaining geometrical parameters are comparable with those of a 5-aniline-substituted thio­hydantoin reported by our group (Kotha *et al.*, 2019 ▸; Cambridge Structural Database refcode FOWGOQ).

**Figure 2:**
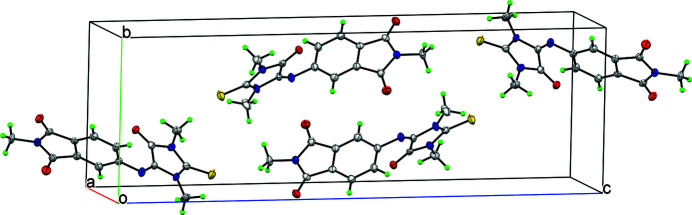
The crystal packing of **1**, viewed along the *a*-axis direction.

The mol­ecular structure of **1** has an angular shape and the mean planes defined by the C10–C12/N1/N2 imidazole ring and C1–C9/N4 pthalimide ring system subtend a dihedral angle of 73.84 (17)°. The bond angle of the C8—N3—C10 linker , which connects the thio­hydantoin ring with the *N*-phenyl substituent is 120.6 (3)°, some 4° less than the corresponding angle in FOWGOQ (Fig. 2 ▸).

The N1 and N2 nitro­gen atoms in the imidazole ring are protected by methyl groups, which rules out the possibility of classical hydrogen bonding in the packing (Fig. 2 ▸), but several weak C—H⋯O links occur (Table 1 ▸).

## Synthesis and crystallization

**Figure 3:**
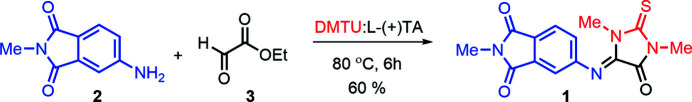
Synthesis scheme for **1**

Initially, a deep eutectic mixture was obtained by mixing di­methyl­thio­urea and l-tartaric acid (DMTU:L–(+)TA) in 70:30 ratio at 80°C. After obtaining the melt, aniline 2 (100 mg, 0.57 mmol) and ethyl­glyoxalate 3 (0.12 ml, 1.14 mmol) were added and the mixture was stirred at the same temperature for 6 h. After completion of the reaction (TLC monitoring), the product was concentrated and purified by silica-gel column chromatography using petroleum ether and ethyl acetate as the eluent to afford the title compound **1** (Fig. 3 ▸). Yellow plates were recrystallized from chloro­form solution (Kotha *et al.*, 2019 ▸).

Yield 108 mg, 60%, m.p. 268–270°C, *R_f_* = 0.76 (60% EtOAc–petroleum ether),^1^H NMR (500 MHz, CDCl_3_) δ 7.79 (*d*, *J* = 7.5 Hz, 1H), 7.38 (*d*, *J* = 2.0 Hz, 1H), 7.20 (*dd*, *J* = 8.0, 1.5 Hz, 1H), 3.42 (*s*, 3H), 3.15 (*s*, 3H), 3.03 (*s*, 3H) p.p.m., ^13^C NMR (125 MHz, CDCl3) δ 180.7, 168.3, 168.3, 154.3, 151.9, 141.4, 133.7, 128.3, 125.3, 124.1, 115.3, 29.6, 28.1, 24.2 p.p.m., HRMS (ESI) calculated for C_14_H_12_N_4_NaO_3_S [*M* + Na] 339.0522, found 339.0526, IR (neat) 3376, 3028, 1767, 1749, 1738, 1712, 1615, 1405, 1383 cm^−1^.

## Refinement

Crystal data, data collection and structure refinement details are summarized in Table 2 ▸.

## Supplementary Material

Crystal structure: contains datablock(s) I. DOI: 10.1107/S2414314621003229/hb4377sup1.cif

Structure factors: contains datablock(s) I. DOI: 10.1107/S2414314621003229/hb4377Isup2.hkl

Supporting information file. DOI: 10.1107/S2414314621003229/hb4377Isup3.cml

CCDC reference: 1847293

Additional supporting information:  crystallographic information; 3D view; checkCIF report

## Figures and Tables

**Table 1:** Hydrogen-bond geometry (Å, °)

*D*—H⋯*A*	*D*—H	H⋯*A*	*D*⋯*A*	*D*—H⋯*A*
C5—H5*C*⋯O2^i^	0.98	2.60	3.519 (5)	157
C7—H7⋯O3^ii^	0.95	2.37	3.302 (4)	166
C9—H9⋯O1^iii^	0.95	2.47	3.319 (4)	149
C14—H14*B*⋯O2^iv^	0.98	2.32	3.272 (5)	164

Symmetry codes: (i) 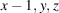 ; (ii) 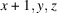 ; (iii) 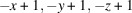 ; (iv) 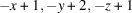 .

**Table 2:** Experimental details

Crystal data	
Chemical formula	C_14_H_12_N_4_O_3_S
*M*_r_	316.34
Crystal system, space group	Monoclinic, *P*2_1_/*n*
Temperature (K)	150
*a*, *b*, *c* (Å)	5.4887 (9), 9.2470 (12), 27.457 (2)
β (°)	94.75 (1)
*V* (Å^3^)	1388.7 (3)
*Z*	4
Radiation type	Mo *K*α
μ (mm^−1^)	0.25
Crystal size (mm)	0.31 × 0.27 × 0.22

Data collection	
Diffractometer	Rigaku Oxford Diffraction Saturn724+
Absorption correction	Multi-scan (*CrysAlis PRO*; Rigaku OD, 2015 ▸)
*T*_min_, *T*_max_	0.340, 1.000
No. of measured, independent and observed [*I* > 2σ(*I*)] reflections	8048, 2343, 1624
*R*_int_	0.105
(sin θ/λ)_max_ (Å^−1^)	0.595

Refinement	
*R*[*F* ^2^ > 2σ(*F* ^2^)], *wR*(*F* ^2^), *S*	0.061, 0.153, 1.04
No. of reflections	2343
No. of parameters	202
H-atom treatment	H-atom parameters constrained
Δρ_max_, Δρ_min_ (e Å^−3^)	0.38, −0.35

Computer programs: *CrysAlis PRO* (Rigaku OD, 2015 ▸), *SHELXT* (Sheldrick, 2015*a* ▸), *SHELXL* (Sheldrick, 2015*b* ▸) and *OLEX2* (Dolomanov *et al.*, 2009 ▸).

## full crystallographic data

### Crystal data

**Table:**

C_14_H_12_N_4_O_3_S	*F*(000) = 656
*M**_r_* = 316.34	*D*_x_ = 1.513 Mg m^−^^3^
Monoclinic, *P*2_1_/*n*	Mo *K*α radiation, λ = 0.71073 Å
*a* = 5.4887 (9) Å	Cell parameters from 2419 reflections
*b* = 9.2470 (12) Å	θ = 2.3–31.0°
*c* = 27.457 (2) Å	µ = 0.25 mm^−^^1^
β = 94.75 (1)°	*T* = 150 K
*V* = 1388.7 (3) Å^3^	Plate, yellow
*Z* = 4	0.31 × 0.27 × 0.22 mm

### Data collection

**Table:**

Rigaku Oxford Diffraction Saturn724+ diffractometer	2343 independent reflections
Radiation source: fine-focus sealed X-ray tube, Enhance (Mo) X-ray Source	1624 reflections with *I* > 2σ(*I*)
Graphite monochromator	*R*_int_ = 0.105
Detector resolution: 28.5714 pixels mm^-1^	θ_max_ = 25.0°, θ_min_ = 2.3°
ω scans	*h* = −6→6
Absorption correction: multi-scan (CrysalisPro; Rigaku OD, 2015)	*k* = −9→10
*T*_min_ = 0.340, *T*_max_ = 1.000	*l* = −32→32
8048 measured reflections	

### Refinement

**Table:**

Refinement on *F*^2^	Primary atom site location: dual
Least-squares matrix: full	Hydrogen site location: inferred from neighbouring sites
*R*[*F*^2^ > 2σ(*F*^2^)] = 0.061	H-atom parameters constrained
*wR*(*F*^2^) = 0.153	*w* = 1/[σ^2^(*F*_o_^2^) + (0.0624*P*)^2^ + 0.3068*P*] where *P* = (*F*_o_^2^ + 2*F*_c_^2^)/3
*S* = 1.04	(Δ/σ)_max_ < 0.001
2343 reflections	Δρ_max_ = 0.38 e Å^−^^3^
202 parameters	Δρ_min_ = −0.34 e Å^−^^3^
0 restraints	

### Special details

**Table:**

Geometry. All esds (except the esd in the dihedral angle between two l.s. planes) are estimated using the full covariance matrix. The cell esds are taken into account individually in the estimation of esds in distances, angles and torsion angles; correlations between esds in cell parameters are only used when they are defined by crystal symmetry. An approximate (isotropic) treatment of cell esds is used for estimating esds involving l.s. planes.

### Fractional atomic coordinates and isotropic or equivalent isotropic displacement parameters (Å^2^)

**Table:**

	*x*	*y*	*z*	*U*_iso_*/*U*_eq_	
S1	0.6276 (2)	0.57457 (11)	0.24925 (3)	0.0309 (3)	
O3	0.5648 (5)	0.8470 (2)	0.40427 (8)	0.0257 (7)	
O2	0.9716 (5)	0.9678 (3)	0.63093 (8)	0.0257 (7)	
O1	0.4516 (5)	0.6022 (3)	0.57964 (8)	0.0316 (7)	
N4	0.6704 (5)	0.7946 (3)	0.61393 (9)	0.0209 (7)	
N2	0.5470 (6)	0.7361 (3)	0.32835 (9)	0.0221 (7)	
N1	0.8657 (6)	0.5885 (3)	0.33889 (9)	0.0220 (7)	
N3	1.0073 (6)	0.6355 (3)	0.41971 (9)	0.0248 (8)	
C4	0.8759 (7)	0.8769 (4)	0.60409 (11)	0.0207 (9)	
C3	0.9437 (7)	0.8266 (4)	0.55580 (11)	0.0203 (9)	
C2	0.7823 (7)	0.7184 (4)	0.53916 (11)	0.0227 (9)	
C10	0.8555 (7)	0.6584 (4)	0.38396 (11)	0.0213 (9)	
C1	0.6097 (7)	0.6926 (4)	0.57754 (11)	0.0216 (9)	
C12	0.6818 (7)	0.6322 (4)	0.30574 (11)	0.0214 (9)	
C11	0.6395 (7)	0.7596 (4)	0.37603 (11)	0.0221 (9)	
C8	0.9789 (7)	0.7009 (4)	0.46559 (11)	0.0250 (9)	
C5	0.5476 (7)	0.8074 (4)	0.65885 (11)	0.0289 (10)	
H5A	0.664931	0.840036	0.685427	0.043*	
H5B	0.481137	0.713115	0.667237	0.043*	
H5C	0.414137	0.877738	0.654078	0.043*	
C7	1.1494 (7)	0.8039 (4)	0.48344 (11)	0.0268 (10)	
H7	1.279648	0.829387	0.464394	0.032*	
C9	0.7927 (7)	0.6529 (4)	0.49413 (11)	0.0247 (9)	
H9	0.680360	0.579570	0.483004	0.030*	
C6	1.1326 (7)	0.8698 (4)	0.52850 (11)	0.0238 (9)	
H6	1.246289	0.941822	0.540186	0.029*	
C14	0.3422 (7)	0.8152 (4)	0.30389 (12)	0.0284 (10)	
H14A	0.403902	0.886751	0.281610	0.043*	
H14B	0.251819	0.864661	0.328326	0.043*	
H14C	0.233247	0.747504	0.285278	0.043*	
C13	1.0409 (8)	0.4761 (4)	0.33055 (13)	0.0360 (11)	
H13A	1.175975	0.479863	0.356201	0.054*	
H13B	1.104667	0.490714	0.298611	0.054*	
H13C	0.960755	0.381459	0.331176	0.054*	

### Atomic displacement parameters (Å^2^)

**Table:**

	*U*^11^	*U*^22^	*U*^33^	*U*^12^	*U*^13^	*U*^23^
S1	0.0393 (8)	0.0339 (7)	0.0201 (5)	0.0062 (5)	0.0060 (4)	−0.0072 (4)
O3	0.0335 (18)	0.0206 (15)	0.0247 (12)	0.0004 (12)	0.0136 (12)	−0.0036 (11)
O2	0.0278 (17)	0.0253 (15)	0.0248 (12)	−0.0008 (12)	0.0064 (11)	−0.0070 (11)
O1	0.0362 (19)	0.0289 (16)	0.0305 (13)	−0.0074 (14)	0.0077 (13)	−0.0029 (12)
N4	0.024 (2)	0.0215 (17)	0.0180 (13)	0.0009 (14)	0.0059 (13)	−0.0003 (12)
N2	0.025 (2)	0.0213 (17)	0.0200 (14)	0.0033 (14)	0.0048 (13)	−0.0030 (13)
N1	0.028 (2)	0.0238 (18)	0.0148 (13)	0.0072 (14)	0.0045 (14)	−0.0038 (12)
N3	0.032 (2)	0.0265 (19)	0.0169 (14)	0.0020 (15)	0.0070 (15)	−0.0017 (13)
C4	0.024 (2)	0.017 (2)	0.0221 (17)	0.0047 (16)	0.0056 (17)	−0.0017 (16)
C3	0.026 (2)	0.016 (2)	0.0191 (16)	0.0056 (16)	0.0027 (16)	0.0020 (14)
C2	0.028 (3)	0.022 (2)	0.0189 (16)	0.0036 (17)	0.0056 (16)	0.0011 (15)
C10	0.027 (2)	0.019 (2)	0.0197 (17)	0.0010 (17)	0.0116 (17)	−0.0003 (15)
C1	0.024 (2)	0.023 (2)	0.0182 (16)	0.0041 (18)	0.0024 (16)	0.0017 (15)
C12	0.023 (2)	0.017 (2)	0.0257 (17)	0.0012 (16)	0.0101 (17)	0.0025 (15)
C11	0.026 (2)	0.019 (2)	0.0225 (16)	−0.0058 (16)	0.0115 (16)	−0.0010 (15)
C8	0.037 (3)	0.021 (2)	0.0177 (16)	0.0078 (18)	0.0074 (17)	0.0006 (15)
C5	0.037 (3)	0.032 (2)	0.0193 (16)	0.0032 (19)	0.0157 (17)	−0.0030 (16)
C7	0.035 (3)	0.026 (2)	0.0208 (17)	0.0046 (19)	0.0112 (17)	0.0000 (16)
C9	0.027 (3)	0.024 (2)	0.0228 (17)	0.0017 (17)	0.0048 (17)	0.0029 (15)
C6	0.026 (3)	0.021 (2)	0.0243 (17)	0.0019 (17)	0.0055 (17)	0.0022 (15)
C14	0.031 (3)	0.026 (2)	0.0290 (18)	0.0074 (18)	0.0048 (17)	0.0010 (17)
C13	0.047 (3)	0.032 (2)	0.0284 (18)	0.020 (2)	0.0041 (19)	−0.0047 (18)

### Geometric parameters (Å, º)

**Table:**

S1—C12	1.644 (3)	C2—C1	1.493 (4)
O3—C11	1.215 (4)	C2—C9	1.382 (4)
O2—C4	1.209 (4)	C10—C11	1.512 (5)
O1—C1	1.210 (4)	C8—C7	1.395 (5)
N4—C4	1.405 (5)	C8—C9	1.410 (5)
N4—C1	1.394 (4)	C5—H5A	0.9800
N4—C5	1.459 (4)	C5—H5B	0.9800
N2—C12	1.390 (4)	C5—H5C	0.9800
N2—C11	1.382 (4)	C7—H7	0.9500
N2—C14	1.458 (5)	C7—C6	1.389 (4)
N1—C10	1.401 (4)	C9—H9	0.9500
N1—C12	1.363 (5)	C6—H6	0.9500
N1—C13	1.448 (4)	C14—H14A	0.9800
N3—C10	1.252 (4)	C14—H14B	0.9800
N3—C8	1.417 (4)	C14—H14C	0.9800
C4—C3	1.481 (4)	C13—H13A	0.9800
C3—C2	1.389 (5)	C13—H13B	0.9800
C3—C6	1.387 (5)	C13—H13C	0.9800
			
C4—N4—C5	123.6 (3)	C7—C8—N3	118.9 (3)
C1—N4—C4	112.1 (3)	C7—C8—C9	121.0 (3)
C1—N4—C5	124.1 (3)	C9—C8—N3	119.9 (3)
C12—N2—C14	124.0 (3)	N4—C5—H5A	109.5
C11—N2—C12	111.4 (3)	N4—C5—H5B	109.5
C11—N2—C14	124.5 (3)	N4—C5—H5C	109.5
C10—N1—C13	123.1 (3)	H5A—C5—H5B	109.5
C12—N1—C10	111.8 (3)	H5A—C5—H5C	109.5
C12—N1—C13	124.9 (3)	H5B—C5—H5C	109.5
C10—N3—C8	120.6 (3)	C8—C7—H7	119.3
O2—C4—N4	125.1 (3)	C6—C7—C8	121.4 (3)
O2—C4—C3	129.4 (3)	C6—C7—H7	119.3
N4—C4—C3	105.5 (3)	C2—C9—C8	116.4 (3)
C2—C3—C4	108.7 (3)	C2—C9—H9	121.8
C6—C3—C4	130.4 (3)	C8—C9—H9	121.8
C6—C3—C2	121.0 (3)	C3—C6—C7	117.5 (3)
C3—C2—C1	107.9 (3)	C3—C6—H6	121.2
C9—C2—C3	122.5 (3)	C7—C6—H6	121.2
C9—C2—C1	129.6 (3)	N2—C14—H14A	109.5
N1—C10—C11	104.3 (3)	N2—C14—H14B	109.5
N3—C10—N1	122.8 (3)	N2—C14—H14C	109.5
N3—C10—C11	132.9 (3)	H14A—C14—H14B	109.5
O1—C1—N4	124.3 (3)	H14A—C14—H14C	109.5
O1—C1—C2	130.1 (3)	H14B—C14—H14C	109.5
N4—C1—C2	105.7 (3)	N1—C13—H13A	109.5
N2—C12—S1	125.7 (3)	N1—C13—H13B	109.5
N1—C12—S1	126.9 (3)	N1—C13—H13C	109.5
N1—C12—N2	107.4 (3)	H13A—C13—H13B	109.5
O3—C11—N2	126.3 (3)	H13A—C13—H13C	109.5
O3—C11—C10	128.5 (3)	H13B—C13—H13C	109.5
N2—C11—C10	105.1 (3)		
			
O2—C4—C3—C2	179.0 (4)	C12—N2—C11—C10	−0.3 (4)
O2—C4—C3—C6	−0.2 (7)	C12—N1—C10—N3	−179.6 (3)
N4—C4—C3—C2	−0.5 (4)	C12—N1—C10—C11	−1.4 (4)
N4—C4—C3—C6	−179.6 (4)	C11—N2—C12—S1	179.7 (3)
N1—C10—C11—O3	−176.8 (3)	C11—N2—C12—N1	−0.6 (4)
N1—C10—C11—N2	1.0 (3)	C8—N3—C10—N1	−175.4 (3)
N3—C10—C11—O3	1.1 (6)	C8—N3—C10—C11	7.1 (6)
N3—C10—C11—N2	178.9 (4)	C8—C7—C6—C3	1.8 (6)
N3—C8—C7—C6	−179.0 (3)	C5—N4—C4—O2	−1.0 (6)
N3—C8—C9—C2	177.7 (3)	C5—N4—C4—C3	178.5 (3)
C4—N4—C1—O1	174.2 (4)	C5—N4—C1—O1	−1.3 (6)
C4—N4—C1—C2	−4.0 (4)	C5—N4—C1—C2	−179.5 (3)
C4—C3—C2—C1	−1.9 (4)	C7—C8—C9—C2	3.1 (5)
C4—C3—C2—C9	177.7 (3)	C9—C2—C1—O1	5.9 (7)
C4—C3—C6—C7	−179.1 (3)	C9—C2—C1—N4	−175.9 (4)
C3—C2—C1—O1	−174.6 (4)	C9—C8—C7—C6	−4.3 (6)
C3—C2—C1—N4	3.6 (4)	C6—C3—C2—C1	177.4 (3)
C3—C2—C9—C8	0.6 (5)	C6—C3—C2—C9	−3.1 (6)
C2—C3—C6—C7	1.9 (5)	C14—N2—C12—S1	−3.3 (5)
C10—N1—C12—S1	−179.0 (3)	C14—N2—C12—N1	176.4 (3)
C10—N1—C12—N2	1.3 (4)	C14—N2—C11—O3	0.6 (5)
C10—N3—C8—C7	−113.3 (4)	C14—N2—C11—C10	−177.2 (3)
C10—N3—C8—C9	72.0 (5)	C13—N1—C10—N3	4.7 (5)
C1—N4—C4—O2	−176.6 (3)	C13—N1—C10—C11	−177.1 (3)
C1—N4—C4—C3	2.9 (4)	C13—N1—C12—S1	−3.4 (5)
C1—C2—C9—C8	−180.0 (4)	C13—N1—C12—N2	176.9 (3)
C12—N2—C11—O3	177.6 (3)		

### Hydrogen-bond geometry (Å, º)

**Table:**

*D*—H···*A*	*D*—H	H···*A*	*D*···*A*	*D*—H···*A*
C5—H5*C*···O2^i^	0.98	2.60	3.519 (5)	157
C7—H7···O3^ii^	0.95	2.37	3.302 (4)	166
C9—H9···O1^iii^	0.95	2.47	3.319 (4)	149
C14—H14*B*···O2^iv^	0.98	2.32	3.272 (5)	164

Symmetry codes: (i) *x*−1, *y*, *z*; (ii) *x*+1, *y*, *z*; (iii) −*x*+1, −*y*+1, −*z*+1; (iv) −*x*+1, −*y*+2, −*z*+1.
